# Accuracy of the HumaSens^plus^ point-of-care uric acid meter using capillary blood obtained by fingertip puncture

**DOI:** 10.1186/s13075-018-1585-0

**Published:** 2018-05-02

**Authors:** Stéphanie Fabre, Pierre Clerson, Jean-Marie Launay, Jean-François Gautier, Tiphaine Vidal-Trecan, Jean-Pierre Riveline, Adam Platt, Anna Abrahamsson, Jeffrey N. Miner, Glen Hughes, Pascal Richette, Thomas Bardin

**Affiliations:** 1 Inserm U1132, Rheumatology Department, Lariboisière Hospital, Paris Diderot University, 2 rue Ambroise Paré, 75010 Paris, France; 2 Soladis Clinical Studies, Roubaix, France; 3 Inserm U942, Biochemistry and Molecular Biology Department, Lariboisière Hospital, Paris Diderot University, Paris, France; 4 Diabetology Department, Lariboisière Hospital, Paris, France; 5 Precision Medicine and Genomics, IMED Biotech Unit, AstraZeneca, Cambridge, UK; 6 Ardea Biosciences, Inc., San Diego, CA USA

**Keywords:** Uric acid, Gout, Point-of-care meter, Capillary blood

## Abstract

**Background:**

The uric acid (UA) level in patients with gout is a key factor in disease management and is typically measured in the laboratory using plasma samples obtained after venous puncture. This study aimed to assess the reliability of immediate UA measurement with capillary blood samples obtained by fingertip puncture with the HumaSens^plus^ point-of-care meter.

**Methods:**

UA levels were measured using both the HumaSens^plus^ meter in the clinic and the routine plasma UA method in the biochemistry laboratory of 238 consenting diabetic patients. HumaSens^plus^ capillary and routine plasma UA measurements were compared by linear regression, Bland-Altman plots, intraclass correlation coefficient (ICC), and Lin’s concordance coefficient. Values outside the dynamic range of the meter, low (LO) or high (HI), were analyzed separately. The best capillary UA thresholds for detecting hyperuricemia were determined by receiver operating characteristic (ROC) curves. The impact of potential confounding factors (demographic and biological parameters/treatments) was assessed. Capillary and routine plasma UA levels were compared to reference plasma UA measurements by liquid chromatography-mass spectrometry (LC-MS) for a subgroup of 67 patients.

**Results:**

In total, 205 patients had capillary and routine plasma UA measurements available. ICC was 0.90 (95% confidence interval (CI) 0.87–0.92), Lin’s coefficient was 0.91 (0.88–0.93), and the Bland-Altman plot showed good agreement over all tested values. Overall, 17 patients showed values outside the dynamic range. LO values were concordant with plasma values, but HI values were considered uninterpretable. Capillary UA thresholds of 299 and 340 μmol/l gave the best results for detecting hyperuricemia (corresponding to routine plasma UA thresholds of 300 and 360 μmol/l, respectively). No significant confounding factor was found among those tested, except for hematocrit; however, this had a negligible influence on the assay reliability. When capillary and routine plasma results were discordant, comparison with LC-MS measurements showed that plasma measurements had better concordance: capillary UA, ICC 0.84 (95% CI 0.75–0.90), Lin’s coefficient 0.84 (0.77–0.91); plasma UA, ICC 0.96 (0.94–0.98), Lin’s coefficient 0.96 (0.94–0.98).

**Conclusions:**

UA measurements with the HumaSens^plus^ meter were reasonably comparable with those of the laboratory assay. The meter is easy to use and may be useful in the clinic and in epidemiologic studies.

## Background

Gout is the most common form of inflammatory arthritis and its incidence has increased over the past decade, with the prevalence now exceeding 1% in most developed countries [1]. Gout causes recurrent attacks of acute arthritis and, if not properly managed, results in destructive arthropathies and kidney damage [2]. It can severely impair the quality of life and represents a significant economic burden by increasing absenteeism and the use of healthcare resources [3]. Gout is also associated with premature death due to frequent comorbidities such as hypertension, type 2 diabetes mellitus, dyslipidemias, obesity, kidney and cardiovascular diseases, and through an independent association with cardiovascular mortality risk [4].

Gout results from chronic monosodium urate (MSU) crystal deposition in and around the joints caused by longstanding hyperuricemia [2]. Thus, an important part of disease management relies on the long-term lowering of uricemia below the MSU saturation point to dissolve pathogenic crystal deposits. Urate-lowering therapies should be targeted to reduce the UA levels below 300 or 360 μmol/l (5.0 or 6.0 mg/dl, respectively), according to the gout severity [5, 6]. The European League Against Rheumatism (EULAR) [6], the British Society for Rheumatology (BSR) [7], and the American College of Rheumatology (ACR) [5] have all produced recommendations for management of the disease that stress the need for a treat-to-target strategy, which is found to be effective in many conditions. Thus, monitoring of uric acid (UA) levels in gout patients is of the utmost importance for successful gout management.

UA is presently routinely measured by a uricase-based laboratory assay on plasma samples obtained after venous puncture [8]. An accurate point-of-care UA meter would allow for rapid and more frequent UA testing by healthcare professionals or self-measurement by patients. Such a device may improve patients’ adherence to urate-lowering therapies, a key factor of management success known to be particularly poor in gout patients [9].

Point-of-care UA meters recently commercialized in Europe allow for immediate UA measurement in capillary blood samples obtained by a fingertip puncture, a method as simple as the commonly used capillary glycemia measurement. A comparison of several of these meters concluded that the HumaSens^plus^ device demonstrated good usability and assay precision, with a coefficient of variation from 4.5% to 8% in five patients who had multiple measurements [10]. The coefficient of determination (*R*^2^) was 0.757 in comparison with liquid chromatography-mass spectrometry (LC-MS), the reference method for UA quantification [11], and 0.806 in comparison with the uricase assay (evaluated in 20 volunteers). The aims of this study were to more broadly assess the reliability of capillary UA measurement by the HumaSens^plus^ point-of-care meter, to determine the best capillary UA thresholds that could be considered predictive of a plasma UA of 360 and 300 μmol/l, and to identify potential confounding factors.

## Methods

### Study design

This was a monocentric cross-sectional study performed at a diabetology day-care center of a French hospital in Paris. The study was approved by the local ethics committee.

### Participants

The patients included in the study were all consecutively seen in the diabetology day-care center between January 2016 and March 2016. All were diabetic, 18 years or older, and were able to understand the requirements and purpose of the study. Patients with gout were not excluded. Patients received a written information sheet with details on the study. All gave informed consent before any study procedure. A subset of patients agreed to sign a specific informed consent for LC-MS measurement. A venous puncture for standard blood biochemistry, which included plasma UA, and a fingertip puncture for capillary glycemia measurement were routinely scheduled in the diabetology day-care center. No supplementary puncture was necessary for this study.

### Data collection

Capillary UA was measured using the HumaSens^plus^ UA meter (European Conformity marked and approved for EU market use only) and compared to a standard UA measurement in the hospital biochemistry laboratory that used a venous sample taken and prepared as Li-Heparin plasma and an Abbott uricase automated colorimetric assay. LC-MS was performed on frozen Li-Heparin samples in AstraZeneca’s Protein Biomarkers Laboratory (Precision Medicine and Genomics, Gothenburg, Sweden). Medical information (demographic characteristics, medication, and co-morbidities) were retrieved from medical files.

### Data analysis

The HumaSens^plus^ meter has a dynamic measurement range of 180–1190 μmol/l (3.0 to 20.0 mg/dl). When the meter read LO or HI, indicating that the individual’s UA value was outside the test range, these values were individually compared to corresponding plasma measurements.

The statistical analysis involved all patients with available capillary and plasma UA measurements. Results are expressed as mean ± SD. Capillary and plasma UA measurements were compared by paired Student *t* test. Differences between methods were calculated for each participant, and the distribution was graphically displayed with kernel density estimation (a nonparametric estimation of density). Agreement between methods was assessed by the intraclass correlation coefficient (ICC) and the Lin’s concordance criterion [12]. When measuring the agreement between pairs of observations, the ICC represents the between-pair variance expressed as a proportion of the total variance of the observations (i.e., the proportion of the total variability in the observations that is due to the differences between pairs). ICC was calculated from a general linear model and was expressed with a two-sided 95% confidence interval (CI). An understanding of Lin’s concordance correlation coefficient is obtained if the line of best fit to the data comparing two methods is shown in a scatterplot plotting the results of one method against the other. The Pearson correlation coefficient provides a measure that describes the extent to which the points in the scatter diagram conform to the best fitting line. Lin’s coefficient modifies the Pearson correlation coefficient by assessing how close the data are about the line of best fit and also how far that line is from the 45-degree line through the origin, this 45-degree line representing perfect agreement. Lin’s coefficient is 1 when all the points lie exactly on the 45-degree line drawn through the origin and diminishes as the points depart from this line and as the line of best fit departs from the 45-degree line [13]. In addition, agreement between methods was assessed by Bland-Altman graphic representation. Confounding factors were searched among age, gender, medications, and biological parameters including fasting glycemia, glycated hemoglobin, creatinine clearance, total cholesterol, low-density lipoprotein cholesterol, high-density lipoprotein cholesterol, triglycerides, and hematocrit in a multiple regression model adjusted on plasma UA with stepwise selection of variables. The best capillary UA thresholds predictive of hyperuricemia (target plasma UA of 360 and 300 μmol/l) were determined with receiver operating characteristic (ROC) curves. To estimate the confounding effect of metformin intake, which was frequent in this population of diabetic patients, the same analyses were repeated in patients with or without metformin intake. Calculations showed that 206 paired measurements were required for an ICC of 80% with precision 0.10 and alpha risk 5%.

Additional analyses were performed in the subset of patients with measurements of HumaSens^plus^ capillary UA, plasma UA determined by uricase automated colorimetric assay, and plasma UA determined by LC-MS. Differences between methods were calculated. ICC and Lin’s concordance coefficient were calculated between each pair of methods. Results for capillary UA and plasma uricase UA were plotted against plasma LC-MS UA results. Concordance between capillary UA and LC-MS UA data and between plasma uricase UA and LC-MS UA data were graphically assessed by the Bland-Altman method. The analysis involved the use of SAS 9.4 (SAS Institute, Cary, NC, USA).

## Results

Two hundred and thirty-eight patients were included in the study. The statistical analysis involved 205 patients after excluding 33 patients with missing capillary or plasma UA measurement (*n* = 16) or with capillary UA outside the HumaSens^plus^ dynamic range (*n* = 17). Patients’ demographic characteristics are presented in Table 1. Most were men (*n* = 134; 65.4%), mean age was 58.1 ± 13.4 years, 151/201 (75.1%) had type 2 diabetes, 140 (68.6%) had hypercholesterolemia, and 120 (58.8%) were hypertensive.

**Table 1:** Characteristics of patients (*n* = 205)

Characteristic	Value
Male gender, *n* (%)	134 (65.4)
Age (years), mean ± SD	58.1 ± 13.4
Body mass index (kg/m^2^), mean ± SD	28.1 ± 5.2
Diabetes type 1, *n* (%)	50/201^a^ (24.9)
Diabetes type 2, *n* (%)	151/201^a^ (75.1)
Coronary disease, *n* (%)	19 (9.3)
Hypercholesterolemia, *n* (%)	140 (68.6)
Hypertriglyceridemia, *n* (%)	32 (15.6)
Hypertension, *n* (%)	120 (58.8)
Myocardial infarction, *n* (%)	7 (3.4)
Stroke, *n* (%)	5 (2.4)
Gout, *n* (%)	4 (2.0)

^a^Type of diabetes was unknown for three patients

### HumaSens^plus^ values outside the dynamic range

Nineteen capillary samples were marked LO by the meter (capillary UA < 180 μmol/l). A second measurement was performed with the meter, and values were obtained for four patients: 180, 180, 270, and 400 μmol/l. Those values were included in the statistical analysis; the other 15 patients were excluded and analyzed separately. Among these 15 patients, 12 had UA values below 180 μmol/l according to plasma measurement, and three had values above: 189, 206, and 428 μmol/l. Two samples were marked HI by the meter (capillary UA > 1190 μmol/l). These were excluded from the statistical analysis and were measured at 303 and 213 μmol/l by the laboratory uricase assay.

### Comparison of HumaSens^plus^ capillary blood and routine plasma UA measurements

**Fig. 1:**
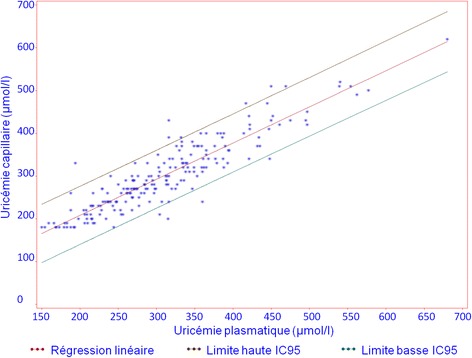
Linear regression analysis comparing HumaSens^plus^ capillary and laboratory uricase-based plasma uric acid (UA) measurements. CI95, 95% confidence interval

Mean capillary blood UA was 300 ± 83 μmol/l (5.04 ± 1.40 mg/dl) and mean plasma UA was 309 ± 90 μmol/l (5.19 ± 1.51 mg/dl). The mean difference between capillary blood and plasma UA was −8.6 ± 36.9 μmol/l (-1.4 ± 6.2 mg/dl; *p* = 0.0010). The linear regression formula was capillary UA = 39.21 + 0.85 × plasma UA (Fig. 1).

**Fig. 2:**
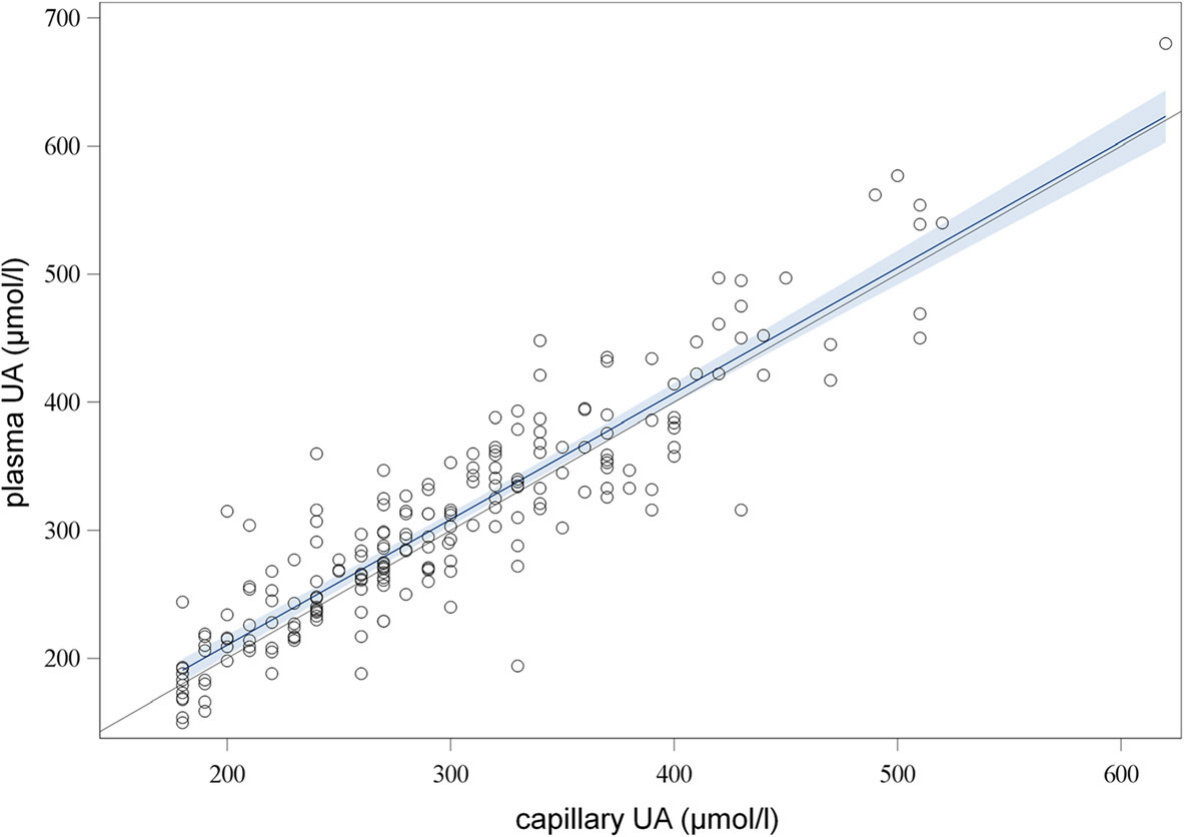
Lin’s concordance correlation coefficient for HumaSens^plus^ capillary and laboratory uricase-based plasma uric acid (UA) measurements. Bold line = linear regression; gray area = 95% confidence level curve fitting; the 45-degree line represents perfect agreement

**Fig. 3:**
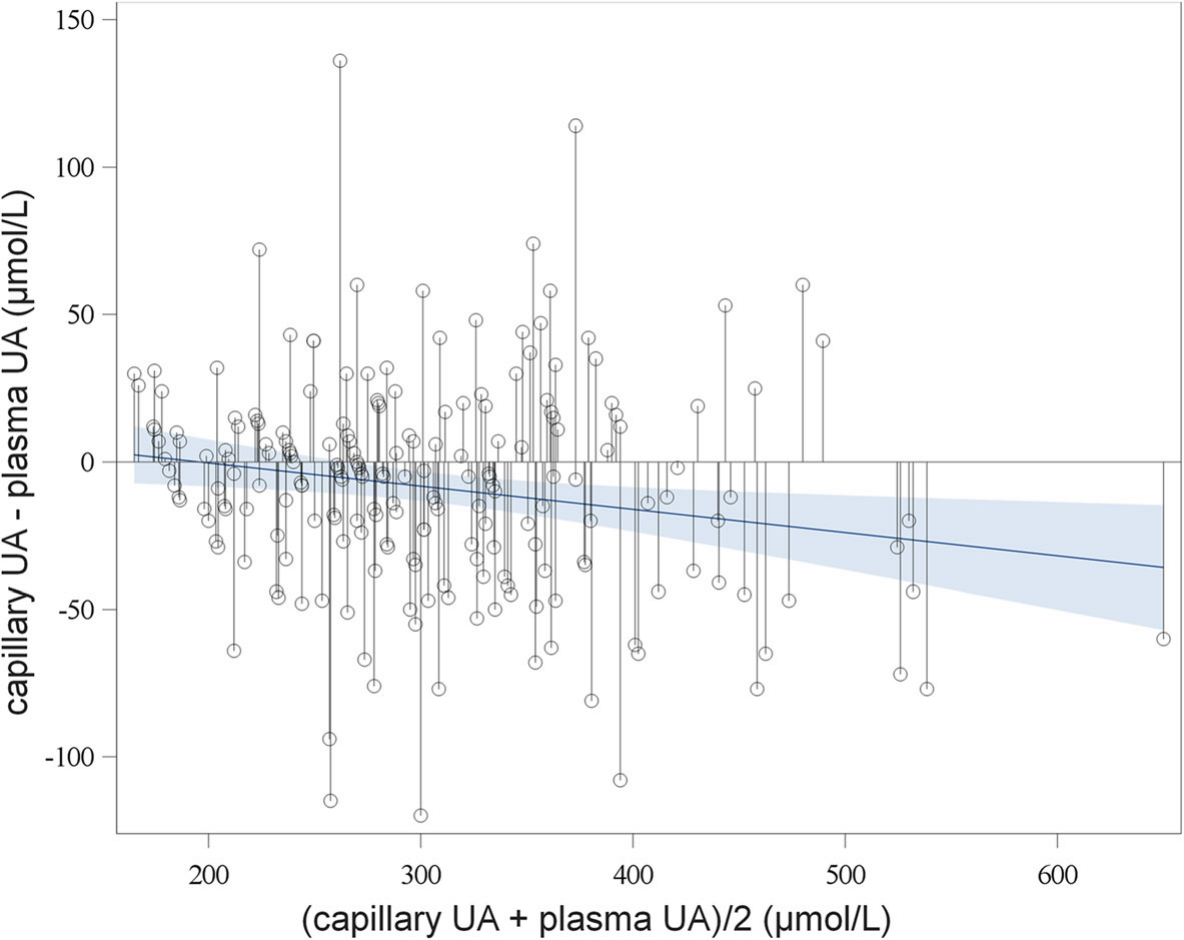
Bland-Altman plot for comparing HumaSens^plus^ capillary and laboratory uricase-based plasma uric acid (UA) measurements. Bold line = linear regression; gray area = 95% confidence level curve fitting

Agreement between capillary blood and plasma UA was high: ICC was 0.90 (0.87–0.92) and Lin’s concordance coefficient was 0.91 (0.88–0.93) (Fig. 2). The Bland-Altman plot (Fig. 3) showed good agreement over all tested values. The estimated bias was 8.6 μmol/l (0.144 mg/dl) with the following 95% limits of agreement: −80.9 μmol/l to 63.7 μmol/l, corresponding to −1.36 to 1.07 mg/dl.

Accuracy of the capillary UA measure with the HumaSens^plus^ meter was 93.2% at the 20% margin.

### Capillary UA thresholds

**Fig. 4:**
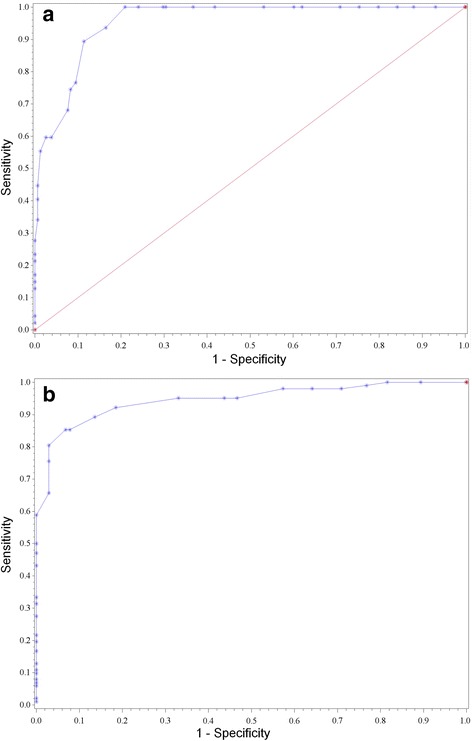
Receiver operating characteristic (ROC) curves of HumaSens^plus^ capillary UA thresholds for plasma UA level **a** ≥ 360 μmol/L and **b** ≥ 300 μmol/L

To detect hyperuricemia with the HumaSens^plus^ meter, defined by a plasma UA level > 360 μmol/l (6.05 mg/dl), the best capillary UA threshold according to the ROC curve was 340 μmol/l, with sensitivity and specificity 89% (Fig. 4a). With the definition of plasma UA level > 300 μmol/l, the therapeutic target UA level for patients with severe gout, the best capillary UA threshold was 299 μmol/l, with sensitivity 85% and specificity 92% (Fig. 4b). Applying the regression formula, a plasma UA threshold of 360 μmol/l corresponded to capillary UA level of 343 μmol/l and a plasma UA threshold of 300 μmol/l corresponded to capillary UA level of 293 μmol/l.

### Confounding factors

Among potential confounding factors, only hematocrit significantly influenced capillary UA measurements (*p* < 0.0001); however, improvement of the determination coefficient when correcting for hematocrit was negligible, from 0.83 to 0.86. The main types of medication patients received are shown in Table 2. No medication appeared to significantly affect the test results. However, although not statistically significant, the difference between capillary and plasma UA measurement seemed higher with than without metformin (−13.6 versus −6.9 μmol/l; *p* = 0.27).

**Table 2:** Main medications taken by patients (*n* = 205)

Medications taken		Number of patients (%)
Oral antidiabetic drug		130 (63.4)
	Biguanide	126 (61.5)
	Sulfonylureas	76 (37.1)
	Other	67 (32.7)
Insulin	Slow	89 (43.4)
	Rapid	92 (44.9)
Antihypertensive drug		118 (57.6)
	Angiotensin-converting enzyme inhibitor	50 (24.4)
	Angiotensin-receptor blocker	55 (26.8)
	Calcium channel blocker	37 (18.0)
	Beta-blocker	32 (15.6)
	Diuretic	43 (21.0)
Lipid-lowering drug		103 (50.2)

### Comparison with LC-MS measurement

**Fig. 5:**
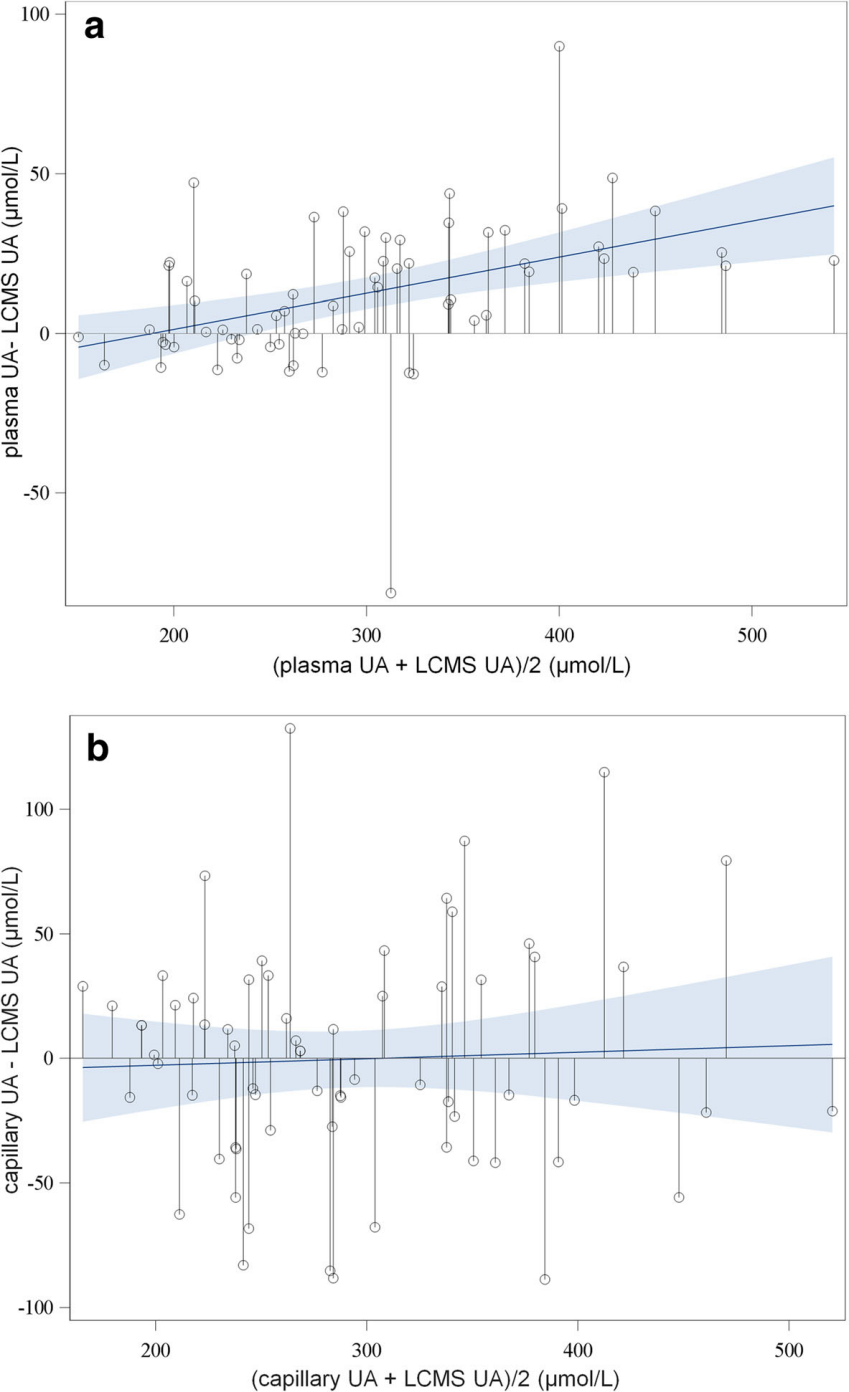
Bland-Altman plot comparing plasma liquid chromatography-mass spectrometry (LC-MS) and **a** plasma uricase-based and **b** HumaSens^plus^ capillary uric acid (UA) measurements. Bold line = linear regression; gray area = 95% confidence level curve fitting

To better understand some discordances between the capillary and routine plasma UA results, these values were compared with plasma UA measurements with the LC-MS reference method in a subgroup of 67 patients (including 24 of 26 with the most discordant results) (Table 3). Agreement between capillary UA and routine plasma UA measurements was acceptable (ICC 0.83 (0.74–0.89), Lin’s concordance coefficient 0.83 (0.76–0.91)). Similar results were found when comparing capillary UA and LC-MS UA measurements (ICC 0.84 (0.75–0.90) and Lin’s concordance coefficient 0.84 (0.77–0.91)). Agreement between routine plasma UA and LC-MS measurement UA was excellent (ICC 0.96 (0.94–0.98) and Lin’s concordance coefficient 0.96 (0.94–0.98)). Bland-Altman plots (Fig. 5) showed that the difference between plasma LC-MS and plasma uricase UA measurements increased with increasing UA level, whereas the difference between plasma LC-MS and capillary UA measurements was independent of the mean UA level, but with a higher dispersion.

**Table 3:** Comparison between LC-MS, uricase-based, and HumaSens^plus^ uric acid (UA) measurements

	Mean ± SD (μmol/l)
Plasma LC-MS UA (*n* = 67)	292.07 ± 80.64
Plasma uricase UA (*n* = 67)	304.46 ± 90.14
HumaSens^plus^ capillary UA (*n* = 67)	291.64 ± 82.60
Δ Plasma LC-MS – plasma uricase UA	−12.39 ± 22.30 (*p* < 0.0001)
Δ Plasma LC-MS – HumaSens^plus^ capillary UA	−0.43 ± 46.19 (*p* = 0.93)

*LC-MS* liquid chromatography-mass spectrometry

## Discussion

In assessing the reliability of immediate UA measurement of capillary blood samples obtained by fingertip puncture, the HumaSens^plus^ meter was easy to use and gave results that were reasonably comparable to those of the uricase laboratory assay within the meter’s dynamic range. Accuracy and agreement with plasma UA measures were good.

A limitation of this study is that the data were obtained from diabetic patients and not gout patients for ethical reasons and ease of implementation since no supplementary puncture was necessary in this population. Thus, the results cannot be generalized to gout patients and therefore we plan to confirm these results in a large population of gout patients.

We found no clinically significant confounding factor for HumaSens^plus^ capillary blood UA measurement among demographic and biological parameters tested or treatments. However, the potential influence of specific gout treatments, too infrequent in this population, could not be assessed. Hematocrit can influence capillary UA measurements by modifying blood viscosity since capillary UA is measured on whole blood. The manufacturer recommends the use of the HumaSens^plus^ meter for a hematocrit range of 30% to 55%. The results of this study confirmed the influence of hematocrit on capillary UA measurements, but it had no real effect on the assay reliability.

In some cases, discordance was observed between capillary and plasma UA measurement. The plasma uricase-based method gave better agreement with the reference method (LC-MS) than capillary UA measurement, especially in those cases. Factors that could affect the capillary UA meter results include tissue perfusion/peripheral blood flow, acid-base status, and temperature. Additionally, according to the manufacturer’s instructions, a cell phone being used in the vicinity could produce potential electronic signal interference. Operator error could also play a role; thus, a second measurement could be considered.

Regarding values outside the meter range, most of the confirmed LO values were concordant with plasma UA; in contrast, the two HI values obtained were not concordant and can be considered uninterpretable until we have further data. A repeated test should also be considered in these cases. However, UA values > 1190 μmol/l, the upper limit of the HumaSens^plus^ range, are very rare in gout patients.

For the target plasma UA levels of 360 and 300 μmol/l, the capillary blood UA thresholds of 340 and 299 μmol/l, respectively, seemed appropriate to discriminate patients with hyperuricemia, the objective of UA measurement in gout.

To improve the results and avoid using capillary UA target threshold values different from the plasma UA threshold commonly used, HumaSens^plus^ devices could be recalibrated by using the linear regression formula capillary UA = 39.21 + 0.85 × plasma UA.

Point-of-care glycemia testing meters have been developed and used for many years, and from 2003 the ISO15197 guideline has established a 20% (% deviation) accuracy requirement in comparison to the reference method to achieve regulatory approval (95% of the measurements must be in the acceptance range) [14]. Recently, this acceptable accuracy was reduced to 15% [15]. Because we lack guidelines for capillary UA measurement, we considered the 20% accuracy boundary to analyze and compare the results with glycemia testing meters. The HumaSens^plus^ meter achieved 93.2% values within a deviation of 20% as compared with plasma uricase measurements.

Good adherence to urate-lowering therapy ranges from only 18% to 44% in gout patients [9], and 20% to 80% of treated patients do not achieve UA target levels [16–18]. Measurement of UA is recommended frequently during the drug titration period (every 2 to 5 weeks for allopurinol use) and twice a year for long-term treatment [5, 6]. A study in the United States and three European countries found that only 2.4% to 41% of patients with a gout diagnosis had at least one UA result available over a 12-month period [16]. Retrospective studies show similarly low rates for patients with at least one UA value available during follow-up [19, 20]. Point-of-care testing allows for easy, immediate, and more frequent UA measurements. Self-measurement by patients could improve their adherence to therapies and recommended dietary and lifestyle changes. With immediate UA measurement during a patient consultation, the general practitioner or rheumatologist could more easily adjust therapies for patients who lack recent laboratory results.

Point-of-care testing and self-management have shown benefits in other clinical areas. Daily self-monitoring of blood glucose has been found to be useful for maintaining glycemic control in noninsulin-treated type 2 diabetes [21], and immediate feedback of HbA1c testing during patient encounters improved glycemic control [22]. Furthermore, self-monitoring of coagulation status in people receiving long-term vitamin K antagonist therapy was found to be cost-effective [23].

Moreover, immediate measurement before pegloticase infusion would facilitate the use of the drug since reinfusions should not be performed in patients with high UA [24]. Point-of-care UA testing would also be interesting in epidemiological studies. With simple measurement and immediately available results, data collection would be easier as laboratory testing is not easily available in the field [25].

## Conclusion

For reliable, immediate UA measurement in capillary blood samples obtained by fingertip puncture, HumaSens^plus^ meters were easy to use and gave results that were reasonably comparable to those of the uricase laboratory assay. HumaSens^plus^-based capillary blood UA measurements can be useful in the clinic and in epidemiologic studies. Further studies are needed to determine the performance of the meter in gout patients and whether frequent UA self-measurement can improve patient adherence to urate-lowering therapies and achievement of target UA levels, especially when combined with educational programs and/or mobile application use.

## Abbreviations

ACR: American College of Rheumatology
BSR: British Society for Rheumatology
CI: Confidence interval
EULAR: European League Against Rheumatism
ICC: Intraclass correlation coefficient
LC-MS: Liquid chromatography-mass spectrometry
MSU: Monosodium urate
ROC: Receiver operating characteristic
UA: Uric acid
